# Enhancing adapted physical activity training for community organizations: co-construction and evaluation of training modules

**DOI:** 10.1093/tbm/ibae065

**Published:** 2024-11-29

**Authors:** Nour Saadawi, Krista L Best, Olivia L Pastore, Roxanne Périnet-Lacroix, Jennifer R Tomasone, Mario Légaré, Annabelle de Serres-Lafontaine, Shane N Sweet

**Affiliations:** Department of Kinesiology and Physical Education, McGill University, 475 Pine Ave W, Montreal, Quebec, Canada; Centre for Interdisciplinary Research in Rehabilitation of Greater Montreal (CRIR), Lindsay Pavilion of the IURDPM, 6363 Hudson Road, Montreal, Quebec, Canada; Centre for Interdisciplinary Research in Rehabilitation and Social Integration (Cirris), Centre Intégré Universitaire de Santé et de Services Sociaux de la Capitale-Nationale (CIUSSS-CN), 525 Wilfrid-Hamel Boulevard, Quebec City, Quebec, Canada; School of Rehabilitation Sciences, Faculty of Medicine, Université Laval, 1050, avenue de la Médecine, Quebec City, Quebec, Canada; Department of Kinesiology and Physical Education, McGill University, 475 Pine Ave W, Montreal, Quebec, Canada; Centre for Interdisciplinary Research in Rehabilitation of Greater Montreal (CRIR), Lindsay Pavilion of the IURDPM, 6363 Hudson Road, Montreal, Quebec, Canada; Adaptavie, 525, boulevard Wilfrid-Hamel Est F-122, Quebec City, Quebec, Canada; School of Kinesiology and Health Studies, Queen’s University, 28 Division St, Kingston, Ontario, Canada; Adaptavie, 525, boulevard Wilfrid-Hamel Est F-122, Quebec City, Quebec, Canada; Centre for Interdisciplinary Research in Rehabilitation and Social Integration (Cirris), Centre Intégré Universitaire de Santé et de Services Sociaux de la Capitale-Nationale (CIUSSS-CN), 525 Wilfrid-Hamel Boulevard, Quebec City, Quebec, Canada; Department of Kinesiology and Physical Education, McGill University, 475 Pine Ave W, Montreal, Quebec, Canada; Centre for Interdisciplinary Research in Rehabilitation of Greater Montreal (CRIR), Lindsay Pavilion of the IURDPM, 6363 Hudson Road, Montreal, Quebec, Canada

**Keywords:** adapted physical activity, kinesiologists, training, community-based organizations, behaviour change, motivational interviewing

## Abstract

Community-based physical activity programmes benefit persons with disabilities. However, there is a lack of evidence-based tools to support kinesiologists’ training in such programmes. This study aimed to co-create and evaluate physical activity training modules for community-based adapted physical activity (APA) programmes. In Phase 1, a working group (*n* = 8) consisting of staff, kinesiologists from two community-based APA programmes, and researchers met over four online meetings to discuss needs, co-create training modules, and assess usability. In Phase 2, a pre–post quasi-experimental design evaluated changes in capability, opportunity, and motivation of kinesiologists (*n* = 14) after completing the training modules, which included standardized mock client assessments and participant ratings of module feasibility. Means and standard deviations were computed for feasibility, followed by paired-samples *t*-tests, along with Hedge’s correction effect size. Mock client sessions underwent coding and reliability assessment. The working group meetings generated two main themes: training in (i) motivational interviewing and behaviour change techniques and (ii) optimizing APA prescription. Nine online training modules were created. In Phase 2, medium to large effects of training modules were observed in capability (Hedge’s *g* = 0.67–1.19) for 8/9 modules, opportunity (Hedge’s *g* = 0.77–1.38) for 9/9 modules, and motivation (Hedge’s *g* = 0.58–1.03) for 6/9 modules. In mock client assessments, over 78% of participants appropriately used five behaviour change techniques and, on average, participants demonstrated good use of motivational interviewing strategies.

The findings indicate that training kinesiologists was feasible and has the potential to enhance community-based physical activity programmes for persons with disabilities.

## Teaser

Modules co-created with community-based organizations improved kinesiologists’ capability, opportunity, motivation, and skills to support persons with physical disabilities in adapted physical activity programmes.

## Graphical Abstract

**Graphical Abstract:**
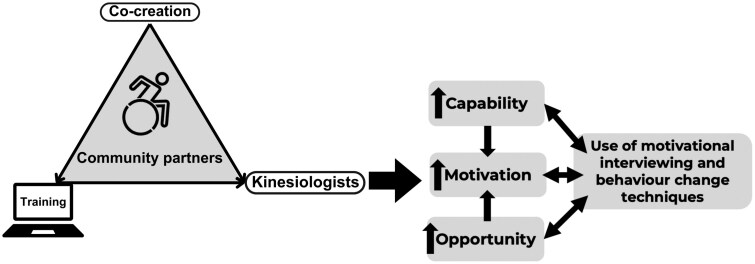

**Implications**

**Practice:** Behaviour change techniques and motivational interviewing modules can be used by kinesiologists to improve their practice with their clientele in community-based organizations for individuals with disabilities.

**Policy:** Adapted physical activity programmes may need to consider behaviour change training modules to improve the practice of kinesiologists.

**Research:** Future research could integrate the modules within organizations to understand adoption, use, and long-term effects on kinesiologists’ behaviours and on members’ physical activity levels and use of behaviour change techniques.

## Introduction

Community-based physical activity organizations deliver programmes that have proven instrumental in enhancing participation, engagement, and health, offering significant benefits for persons with physical disabilities [1]. Nevertheless, institutional barriers often hinder the effectiveness of interventions for persons with disabilities. These barriers include a lack of disability-specific knowledge, minimal physical activity training for professionals, and the limited involvement of these programmes and professionals in the design, testing, and implementation of interventions [2, 3]. Thus, the involvement of community-based organizations in intervention design is a viable solution to help facilitate the uptake of evidence-based practices [4].

Persons with physical disabilities have emphasized the importance of staff knowledge in adapted physical activity (APA)—the adaptations made to physical activity to ensure equitable opportunities for people with disabilities—and their ability to address specific needs within community-based organizations [5], which creates inclusive physical activity environments [6]. However, there is a gap in the training of staff, which emphasizes the need to improve their skills and knowledge to meet the needs of persons with physical disabilities [5, 7]. Staff in APA include kinesiologists, healthcare professionals who have undergone a university degree and specialize in physical activity. While kinesiologists assess, programme, treat, and enhance performance for a diverse range of individuals, including those with physical disabilities, they have indicated a need and desire to obtain evidence-based information and training to perform their current roles [6, 8]. To address these needs, the purpose of this research was to co-create and evaluate physical activity training modules for community-based APA programmes. This research was done in two phases, which are presented separately below.

## Phase 1: Co-construction of Training Modules

Barriers such as limited education, lack of training, and the absence of relevant materials related to persons with physical disabilities underscore the need to develop such training resources for kinesiologists working in APA settings [7]. In fact, community-based organizations have highlighted a need for training resources [6, 8] but the specific content and methods of training delivery are not clear. Further, grounding training in educational theories is both helpful and essential for evidence-based educational practice, facilitating the selection of instructional strategies, learning objectives, assessments, and evaluations [9]. Kolb’s experiential learning theory is an educational framework which posits that learning is a cyclic process comprising four stages: concrete experience, reflective observation, abstract conceptualization, and active experimentation [10]. Recent studies have replicated Kolb’s learning cycle in medical education simulations and training programmes for direct support professionals working with individuals with intellectual disabilities, demonstrating its practical applicability and effectiveness [11, 12]. Using Kolb’s theory as a guide to develop resources, Phase 1 of this study aimed to identify the needs of community-based organizations that offer APA programmes to inform the design and content of training.

### Methods

#### An integrated knowledge translation approach to work in partnership

**Figure 1:**
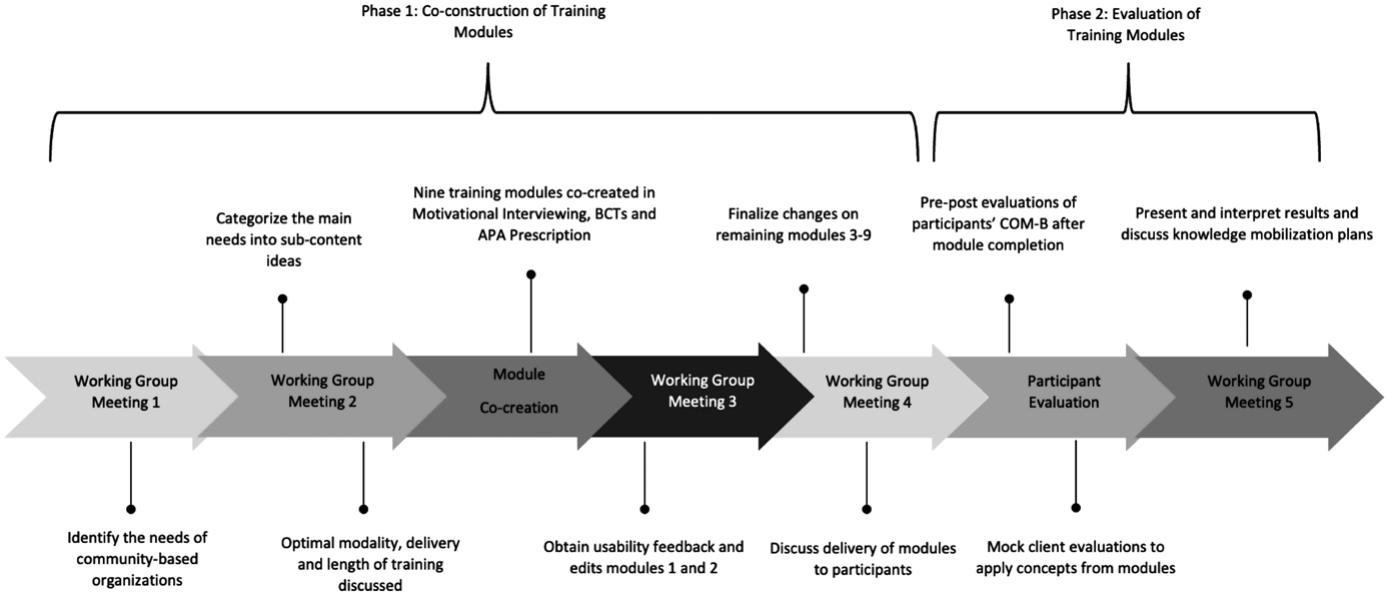
Timeline of Phase 1 and Phase 2 procedures

Adaptavie and Viomax, two medium-sized Québec-based community organizations for adults and youth living with functional limitations, have identified a critical gap in the training of their kinesiologists [5, 8]. The two organizations offer a multitude of APA programmes, sports, and social activities (e.g. physical conditioning, group or individual consultations for social interventions and autonomy, paracycling, rugby, and walking sports clubs). As kinesiologists form a significant portion of the staff at Adaptavie and Viomax, it is crucial to understand their needs to enhance and standardize their professional practice. To build a strong and meaningful partnership with Adaptavie and Viomax, a working group comprised administrative staff (operational managers and programme coordinators), kinesiologists from both organizations, and graduate students and researchers from two universities. The working group met five times, following integrated knowledge translation (IKT) principles to ensure co-creation with partners [13]. The strategies employed by the working group and their alignment with each IKT principle are illustrated in Table A in supplemental materials. Figure 1 presents a timeline of all study procedures.

#### Design, participants, and procedures

The nominal group technique (NGT) was used to provide structure in the first two working group meetings. NGT allows group members to generate a high number of ideas, discuss ideas equally, and rank ideas attentively [14]. Participants of Phase 1 consisted of working group members (*n* = 8), including operational managers, programme coordinators, and practicing kinesiologists from Adaptavie and Viomax. Participants were conveniently and purposively selected because their inclusion was crucial for identifying needs and collaborating on the training modules, ensuring comprehensive organizational representation and effective engagement with the primary knowledge users. The working group meetings were 3 h each and were held on an online meeting platform. Supplemental Materials Text A and Figure A provide a detailed description of Phase 1 procedures and analysis.

**Figure 2:**
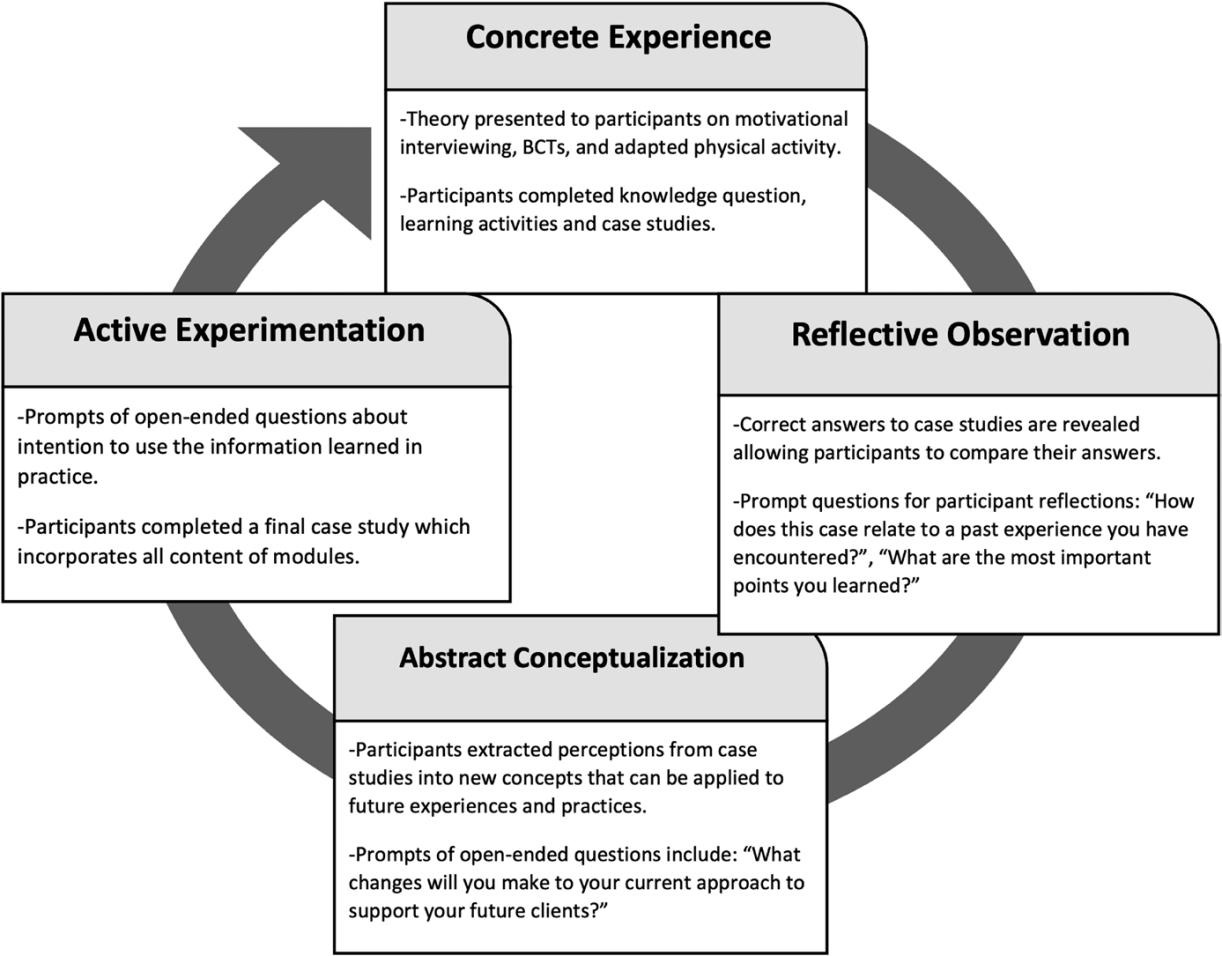
Modules framed by Kolb’s four-stage cycle. *Note.* Adapted from Kolb (1984).

The first working group meeting aimed to identify the general needs of the staff and kinesiologists at Adaptavie and Viomax, such as the content and general themes of the training modules. The second meeting categorized the needed themes into sub-content ideas and discussed the optimal modality, delivery format, and length of training. Based on these meetings, nine online modules were developed with consensus, to frame them using Kolb’s theory, as it was most favoured by the working group. Figure 2 illustrates Kolb’s theory implementation in each module [10], with module content detailed in the results. The third working group meeting evaluated usability and refined the first two training modules. Subsequent module creation incorporated usability feedback, requiring only one round of revisions. The fourth working group meeting aimed to finalize changes on the remaining seven modules and discuss the delivery of modules to participants in Phase 2. The working group meeting results were presented through a descriptive summary. A fifth working group meeting was held to interpret results and plan knowledge mobilization at Adaptavie and Viomax.

### Results and discussion

#### Needs of Adaptavie and Viomax

The first working group meeting generated 59 ideas regarding the needs of the organizations. The NGT voting process facilitated the final selection of 10 ideas and identified two distinct themes: *Theme 1* focused on training in behaviour change techniques (BCTs) and motivational interviewing (MI) to improve and maintain physical activity levels of the clients. *Theme 2* revolved around optimizing APA prescription. Specifically in the first working group meeting, our partners identified the need to enhance kinesiologists’ skills in motivation, effective communication, and actionable techniques. The focus was on strategies for kinesiologists to help clients improve and maintain physical activity levels. Our partners identified that developing MI (i.e. a person-centred communication approach to foster change [15]) skills and learning BCTs (i.e. skills and strategies that are active ingredients of behavioural interventions [16]) like goal setting and social support would be key to this improvement. The identification of these skills by our working group is pertinent as the use of MI and BCTs can enhance the effectiveness of physical activity interventions and contribute to the behaviour change process [15–17]. Common BCTs such as reviewing behaviour goals, self-monitoring, and problem-solving consistently promote physical activity among persons with physical disabilities [2, 18]. Further, kinesiologists trained in MI can effectively guide individuals with disabilities, helping them overcome barriers and achieve their physical activity goals [17, 19]. The second working group meeting resulted in a total of 19 sub-content ideas: 16 ideas related to Theme 1 and 3 ideas related to Theme 2. The 19 ideas were used to inform the creation of the modules. In addition, when discussing the modality of training, the meeting concluded with a consensus to use programmed instruction with interactive case studies, learning activities, and questions.

#### Training modules

The first three training modules focused on MI. The next four modules focused on BCTs, including *Self-monitoring, Goal Setting, Action Planning and Problem Solving,* and *Social Support*. Module 8 was optimizing APA prescription with the use of a physical activity prescription toolkit, and Module 9 consisted of a comprehensive case study that incorporated activities, questions, and reflections based on the information covered in all preceding modules.

#### Module usability

The four categories regarding the usability of the modules were: (i) Structure, clarity, and wording (40 comments), (ii) Positive feedback (27 comments), (iii) Complexity and diversity of case studies (17 comments), and (iv) Role of kinesiologists (8 comments). Based on the feedback regarding the complexity and diversity of case studies, several modifications were made to the modules throughout the editing phases. These changes primarily involved incorporating specific case studies that closely reflect real-life situations that kinesiologists encounter. Adjustments were made to include sidenotes in each module, highlighting the role of kinesiologists with helpful tips explaining their scope of practice.

## Phase 2: Evaluation of Training Modules

### Introduction

Phase 1 highlighted the need for kinesiologists to enhance their skills in MI and BCTs to effectively support clients in improving and maintaining physical activity levels. Phase 1 also highlighted the importance of using Kolb’s theory for effective training delivery. However, while Kolb’s theory provides a strong framework for structuring training, it does not offer specific metrics for assessing changes in kinesiologists’ psychosocial outcomes and their behavioural practices. To evaluate these changes, we need a robust theoretical framework. One such framework that can help us understand and measure the behaviours of kinesiologists is the capability, opportunity, and motivation behavioural model (COM-B) [16].

The COM-B model identifies capability (psychological and physical, such as knowledge and skills), opportunity (physical and social external factors that influence planned behaviour), and motivation (process that energize and direct behaviour, such as goals, habits, and emotions) as the three key components that influence behaviour change [16]. The COM-B model has been applied to evaluate behaviours, train professionals, and develop training interventions [11, 20]. For example, Overwijk *et al*. [11] applied the COM-B model to aid direct support professionals in their interactions with individuals with intellectual disabilities. They evaluated knowledge, skills, and attitudes of direct support professionals, after training and highlighted increased levels of capability and motivation. The training placed a strong emphasis on COM-B determinants and effectively facilitated participant learning by leveraging Kolb’s theory, thereby offering evidence of the synergistic utilization of these two theories [11]. By applying the COM-B model, we can gain a comprehensive understanding of the capability, opportunity, and motivation factors that impact the training of kinesiologists and their ability to promote behaviour change effectively. Therefore, phase 2 of this study was guided by the following research question: what is the effect of the training modules on the capability, opportunity, and motivation of kinesiologists at community-based organizations to promote physical activity among persons with disabilities?

### Methods

A pre–post quasi-experimental design was used to evaluate the changes in capability, opportunity, and motivation as well as physical activity behaviour change counseling of kinesiologists after completing all training modules.

#### Participants and procedures

Eligible participants were enrolled using non-probability convenience sampling. Participants were selected based on availability and interest. This research was approved by the university’s Research Ethics Board (REB #22-07-022). The research team emailed all 15 participants whose name was provided by Adaptavie and Viomax working group members. Eligibility requirements include being a kinesiologist or student of kinesiology, currently employed at Adaptavie or Viomax, and aged 18 years or older. Members of the working group were excluded.

Participants provided informed consent. After completing a baseline questionnaire (demographic information and pre-COM-B questionnaire), participants had access to training modules via LimeSurvey. Participants were asked to complete the training within a 2-week timeframe and completed a feasibility questionnaire after each module. Once all modules were completed, participants completed the post COM-B questionnaire within 1 week. They were then assessed during interactions with a mock client, representative of persons with disabilities, to determine their ability to apply the concepts from the modules and the quality of skills used. The mock client was a person with a spinal cord injury with 37 years of lived experience with APA. The mock client and a researcher co-created an informal script to guide them through the session, with certain adjustments made through meetings and practice. Mock client sessions were recorded via videoconferencing and timed at 15 min/participant.

#### Measures

##### Demographic information.

Participants reported their age, gender, ethnicity, years length of employment, years of experience as a kinesiologist, number of professional education series conducted, certifications, and presence of additional training specific to APA or behaviour change.

##### COM-B.

A COM-B questionnaire consisted of six items, two items for capability (e.g. knowledge and skills, e.g. *“I have the necessary knowledge to use motivational interviewing”*), opportunity (e.g. resources and support), and motivation (e.g. confidence and willingness) with the phrase “to promote physical activity for persons with disabilities” added after each item. Participants completed the questionnaire after all modules were complete. Each item was assessed on a 7-point Likert scale, ranging from 1 (*strongly disagree)* to 7 *(strongly agree).* The COM-B questionnaire was modified based on a previously used brief COM-B questionnaire [21]. For each module, a mean score of the two items for capability, opportunity, and motivation was calculated.

##### Module Feasibility Questionnaire.

Feasibility questionnaire was adapted from Gainforth, Hoekstra [13], to assess six items, including rating the module’s *appeal*, *relevance, appropriate use of language, specificity/unambiguity, acceptability, and potential for unintended adverse effects* on a 7-point Likert scale, ranging from 1 (*strongly disagree),* to 7 *(strongly agree*).

##### Mock Client Coding Sheet.

The coding sheet based on the Motivational Interviewing Treatment Integrity (MITI 4.2.1) scoring tool was developed to code the mock sessions [22]. Each kinesiologists’ counseling utterances were added to the coding sheet as one of the following categories: total MI adherent utterances (seeking collaboration, emphasizing autonomy and affirmations), total MI non-adherent count (persuade and confront), open-ended questions utterances percentage of complex reflections (complex reflections divided by total reflections; where 50% is a good competency and proficiency), and reflection to question ratio (total reflections divided by total questions; where 1:1 ratio is fair and 2:1 is a good competency and proficiency). In addition, overall scores were calculated on a 5-point Likert scale for *cultivating change talk and softening sustain talk (technical global scores), and partnership, and empathy (relational global scores)*, where a 3/5 is considered a fair competency and proficiency threshold, and a 4/5 is considered good. Following a similar coding sheet to the MITI, BCTs were coded by determining opportunities and extent of use on a 5-point Likert scale, where 1 was coded for *(vaguely or unsuccessfully used the BCT),* 5 coded for (*successfully delivered the BCT and guides the client to implement it)* and zero indicated non-used (Figure B, Supplemental Materials).

#### Data analysis

All analyses were conducted using SPSS version 27. Descriptive analysis was used to present sociodemographic information and module feasibility. Means and standard deviations were calculated for the feasibility component of each module, and a total mean was computed. Paired-samples *t*-tests were conducted to evaluate the impact of each training module on participants’ capability, opportunity, and motivation to promote physical activity for persons with disabilities. A Hedge’s correction effect size was calculated using the paired-sample *t*-test option, along with the corresponding confidence intervals [23]. Effect sizes of 0.2 were considered small, 0.5 medium, and 0.8 large.

Two coders used the MITI coding manual to rate kinesiologists’ adherence to the training protocol in the mock client evaluation. A practice coding session was conducted where each session was coded independently. Reliability between the two coders for MI (behaviour counts and overall scores) and BCTs (scale) were compared using intraclass correlation (ICC2) estimates based on a single-measures, consistency, two-way mixed-effects model (0.5–0.75: moderate; 0.75–0.90: good; 0.90+: excellent reliability). BCTs (opportunity to use) were compared with interrater reliability (Cohen’s Kappa coefficient). After initial reliability analysis, coders reviewed codes for one participant, resolved discrepancies, and independently recoded mock client sessions. Interrater reliability and intraclass correlations were conducted again.

## Results

Of the 15 eligible participants contacted, 14 completed all modules (see Table 1 for demographic information). Participants self-reported spending a mean of 32 minutes per module (range: 10–120 minutes), representing an average of 285 minutes to complete all nine modules. Across all nine modules, participants agreed to strongly agree that the modules were feasible (with self-monitoring perceived as less appealing), and no modules were perceived to have unintended adverse effects (Table B, Supplemental Materials).

**Table 1:** Socio-demographic characteristics of participants in phase 2*.*

Variables	*N* (*n* = 14)	
Age (years)		
20–25	9	
>25	5	
Language^*^		
French	14	
English	4	
Arabic	1	
Gender		
Male	5	
Female	9	
Ethnicity*		
Arab	1	
Southeast Asian	1	
White	12	
Education^a^		
High School Diploma or some college/cegep/university	5	
College/cegep diploma or certificate	4	
University degree	12	
Role*		
Kinesiologist	9	
Intern or student	5	
Administrative staff	2	
Year since certification		
<1 year	1	
>1 year	5	
Received additional training		
Yes	8	
No	6	
Training type (if yes to above)		
University course training	3	
Organizational training	5	
Variable	Mean	Range (years)
Years at Adaptavie and Viomax	4.04	0–16

^a^Participants were able to select more than one option. Participants had the option to not answer sociodemographic questions.

COM-B questionnaire descriptive and statistical information are reported in Table 2. For MI, there were medium to large increases in capability, opportunity, and motivation. For action planning, self-monitoring, and social support, participants reported medium to large increases in capability, opportunity, and motivation. For goal setting, only increases in opportunity were reported, while increases in capability and opportunity were found for problem-solving and APA prescription modules.

**Table 2:** Pre–Post COM-B of motivational interviewing, adapted physical activity prescription and BCTs

Training modules	COM-B	Pre		Post		*t* (13)	*P*	Hedge’s g	CI (95%)
		Mean	SD	Mean	SD				
Motivational Interviewing	Capability	5.39	1.11	6.18	0.54	−3.56	.003	0.92	[1.53, 0.29]
	Opportunity	5.00	1.21	5.96	0.80	−2.82	.014	0.73	[1.30, 0.14]
	Motivation	5.57	1.11	6.14	0.60	−2.23	.044	0.58	[1.12, 0.16]
Self-monitoring	Capability	4.07	1.74	6.21	0.61	−4.19	.001	1.09	[1.73, 0.42]
	Opportunity	4.04	1.15	5.93	0.55	−5.31	.000	1.38	[2.10, 0.64]
	Motivation	4.71	1.48	6.11	0.68	−3.95	.002	1.03	[1.66, 0.37]
Goal setting	Capability	6.04	0.66	6.25	0.64	−1.03	.321	0.77	[1.34, 0.17]
	Opportunity	5.18	1.15	6.07	0.55	−2.96	.011	0.27	[−0.78, 0.26]
	Motivation	6.18	0.61	6.36	0.69	−1.10	.292	0.29	[−0.80, 0.24]
Action planning	Capability	5.57	0.96	6.25	0.47	−2.72	.017	0.71	[1.27, 0.12]
	Opportunity	4.96	0.91	6.04	0.57	−4.19	.001	1.09	[1.73, 0.42]
	Motivation	5.68	1.01	6.25	0.33	−2.39	.033	0.62	[1.17, 0.49]
Problem-solving	Capability	5.54	1.01	6.18	0.42	−2.59	.022	0.67	[1.23, 0.93]
	Opportunity	5.00	1.07	6.07	0.65	−4.02	.001	1.04	[1.68, 0.39]
	Motivation	6.00	0.56	6.25	0.55	−1.84	.089	0.48	[−1.01, 0.72]
Social support	Capability	5.00	1.13	5.93	0.68	-4.60	.001	1.19	[1.86, 0.50]
	Opportunity	4.75	1.31	6.00	0.48	−4.53	.001	1.18	[1.84, 0.49]
	Motivation	5.71	0.85	6.14	0.50	−2.92	.012	0.76	[1.33, 0.16]
Adapted Physical Activity Prescription	Capability	5.79	0.83	6.32	0.67	−2.60	.022	0.67	[1.23, 0.95]
	Opportunity	5.68	0.70	6.46	0.60	−3.56	.003	0.92	[1.53, 0.29]
	Motivation	6.18	0.67	6.46	0.46	−1.85	.088	0.48	[−1.01, 0.07]

Negative *t* values indicate higher means for post data compared to pre.

Nine mock client sessions were recorded with a total of 309 utterances (five participants never responded to emails to schedule the sessions). All nine participants had an opportunity to use goal setting, problem-solving and social support, eight participants had an opportunity to use action planning, and three participants had an opportunity to use self-monitoring during the mock client sessions. When opportunities presented themselves, 100% of participants used self-monitoring, action planning and problem-solving, 89% of participants used goal setting, and 78% of participants used social support. For the quality of BCT use, moderate to high-quality ratings (3+ out of 5) were found for action planning (62.5% of participants), problem-solving (77.8%), and social support (57.1%), while low ratings were coded for most participants on self-monitoring (66.7%) and goal setting (87.5%). See Table 3 for the quality of ratings per BCT.

**Table 3:** Motivational interviewing overall scores and BCT use and quality: mock client sessions

Motivational interviewing overall scores	*n* (fair-good cut-off)	Mean	SD	Median	Minimum	Maximum	ICC	
Technical global^a^	9	4.00	0.56	4.00	3	4.5	0.73	
Relational global^a^	7	4.11	0.99	4.50	2	5	0.88	
%CR^a^	7	53.6%	25.4%	50.0%	0.00%	90.0%	0.91	
R to Q ratio^a^	0	0.53	0.26	0.50	0.20	1.11	0.94	
% Open-ended Q^a^	-	35.0%	18.0%	36.4%	0.00%	61.5%	-	
Total MI adherent	-	5.11	1.36	5.00	3	7	0.92	
Total MI non-adherent	-	1.67	1.73	1.00	0	5	0.85	

BCT use and quality	n	N^b^ (used)	Mean (SD)	Median	Minimum	Maximum	Kappa	ICC
Self-monitoring	3	3	2.33 (1.53)	2.00	1	4	1	-
Goal setting	9	8	2.00 (0.54)	2.00	1	3	1	0.76
Action planning	8	8	3.25 (1.17)	3.50	2	5	-	0.88
Problem-solving	9	9	3.11 (1.05)	3.00	1	4	1	0.94
Social support	9	7	2.43 (0.79)	3.00	1	3	1	0.95

*n* = 9.

^a^Technical Global scores cutoffs are: 3/5 (fair), 4/5 (good). Relational Global scores cutoffs are 3.5/5 (fair), 4/5 (good). %CR is the complex reflection percentage, with cutoffs of 40% for fair and 50% for good. R to Q Ratio denotes the reflection to question ratio, with 1:1 ratio for fair, and 2:1 for good. %Open-ended Q is open-ended questions.

^b^Participants who used the BCT when opportunity was presented. Action planning Kappa and self-monitoring ICC cannot be analysed due to constant values and small sample size.

The sessions ranged between 12 and 17 minutes (mean = 15:53) with the full session time being coded. Across all mock client sessions, the coders had moderate to excellent reliability for MI overall scores and behaviour counts (ICC = 0.73–0.94; Table 3). Across all modules, coders had excellent reliability for BCTs opportunity and good to excellent reliability for BCTs scale (ICC= 0.76-0.95). Self-monitoring did not have enough data to conduct proper analysis (Table 3).

All nine participants demonstrated fair to good (4.00 out of 5) MI technical global scores and 7/9 participants demonstrated good relational global scores with score of at least 4.5. Of the nine participants, seven had good complex reflections percentages (competency and proficiency of 53.6%) and the two others scored 37.5%, and 0%. However, participants reflection to question ratio was low, and 35% of participants’ questions were open-ended. Participants used adherent MI language three times more than non-adherent MI language (Table 3). Participants used more persuasian with permission utterances than persuasian without permission utterances and avoided confrontation while using an average of eight adherent language utterances (Table C, Supplemental Materials).

## Discussion

The objective of this study was to co-create and evaluate training resources for kinesiologists at community-based organizations. The training enhanced staff reported capability, opportunity, and motivation to use MI, BCTs, and APA prescription to promote physical activity among persons with disabilities. Our results address an existing gap in evidence-based tools for training kinesiologists by emphasizing co-creation with community partners and combining two theoretical frameworks. Additionally, the findings provide valuable practical insights for enhancing staff training, improving programme effectiveness and sustainability, and enhancing accessibility to APA programmes.

The success of Phase 1 was largely attributed to the strong partnership between researchers and community-based organizations’ staff, guided by IKT principles [13]. Notably, techniques such as NGT and a shareable whiteboard fostered open communication, built rapport, and facilitated shared decision-making. This inclusive approach addressed limitations seen in other online behaviour change interventions [24]. This co-creation process resulted in highly relevant training modules that met partners’ needs, making this research relevant and useful.

The working group’s identified needs, including lack of client motivation and kinesiologists’ insufficient training in MI and BCTs, resonate with recent studies emphasizing this training necessity [5, 6, 25]. These needs support the significance of addressing institutional barriers, like professionals’ lack of disability-specific knowledge [3]. Thus, this study fills a significant gap in evidence-based training resources, offering novel and standardized resources for kinesiologists that meet the specific needs of community-based APA programmes.

Combining MI and BCTs is particularly important to enhance the practice of kinesiologists for APA behaviour change [7, 19]. The limited application of BCTs and MI in fitness centres may be attributed to the predominant emphasis on physical activity prescription in kinesiology curriculum [24, 26]. Our approach of incorporating MI throughout all modules effectively addresses this gap and may partly explain why capability, opportunity, and motivation increased across most modules. By considering physical activity prescription guidelines along with motivation, client knowledge, and the role of kinesiologists [26, 27], our approach fills the curriculum’s lack of content and yields positive outcomes.

Kolb’s adult learning theory is an applicable framework as it guided module delivery, addressed structural needs, and enhanced participant learning. Usability feedback emphasized the value of complex case studies and the kinesiologists’ role in ambiguous situations, which is in line with Kolb’s emphasis on hands-on learning. High feasibility ratings and positive changes in capability, opportunity, and motivation, along with significant MI usage, support the integration of Kolb’s theory into kinesiologists’ training and its potential impact on their professional development. Although scarce, previous interventions utilizing Kolb’s theory showed positive impacts on professional development [11, 12] but lacked evaluation of psychosocial processes and content usage. Our study filled this gap, using the COM-B model for evaluation.

Our study aligns with previous research utilizing the COM-B model for behaviour change training programmes [11, 28]. Overwijk*et al*. [11] and Hoekstra *et al.* [28] applied the COM-B model to assess BCTs and professional roles’ impact on professionals’ knowledge, skills, and attitudes, both showing increases consistent with our findings. Unlike prior studies, our unique focus was on evaluating kinesiologists’ COM-B before and after each training module, allowing for proficiency assessment in specific areas. The potential effectiveness of the modules demonstrates within-participant improvements in COM-B, with varying extents across modules. Notably, capability in social support showed the highest increase, while opportunity saw the highest increase overall, and motivation increased for MI, action planning, social support, and self-monitoring. Capturing the unique changes in COM-B across modules enables a deeper interpretation of the impact of each module on various COM-B components.

The consistent increases in social support across COM-B constructs may be because it is a new BCT concept for kinesiologists. While previous studies did not explicitly train professionals in social support, they observed improvements in BCTs with similarities, such as providing health information and instructions on how to perform behaviours [11]. Further, the increase in opportunity across all modules may be attributed to our partners’ efforts in addressing opportunity barriers. Strategies included allowing staff to complete the training during work hours and emphasized the organizational benefits. These strategies are crucial because professionals often face financial constraints, time limitations, and training restrictions, which restrict opportunities for change [7, 20]. These results underscore the potential for increase efficacy when resources are co-created with end-uses, rendering meaningful outcomes for all partnership members.

Further, the small change observed in capability and motivation for goal setting and motivation for problem-solving and APA prescription may be due to participants’ pre-existing high level of proficiency, potentially influenced by their perceived skill levels [29]. The inherent practice of kinesiologists to prescribe physical activity and offer solutions to client barriers may explain their previous motivation in APA prescription and problem-solving [2]. Nonetheless, we found increases in motivation in self-monitoring, action planning, and social support, which supports the need to assess motivation. In fact, previous studies have primarily focused on enhancing professionals’ capability while neglecting or inadequately addressing motivation, despite its crucial role [25]. Measuring all three aspects of COM is therefore important to fully understand the impact of training resources and thus is recommended for future studies.

From a behavioural perspective, the mock client results in MI align with established behaviour count cutoffs and global scores from MITI 4.2.1. A study evaluating MI in clinical practice also found positive outcomes from using adherent language, encouraging change talk, redirecting sustain talk, fostering partnership, and showing empathy [30]. Similarly, the low ratio of reflections to questions observed in our study aligns with findings from previous research and can be attributed to the evaluative nature of kinesiologists’ sessions [30]. Given participants had 15 minutes with the mock client, they asked numerous questions about the client’s activities, goals, and general health, which contributed to the high frequency of questions being coded. It is important to note that achieving proficiency in MI skills requires practice and tends to decay over time [30]. However, while the modules served as an effective introduction to MI, we recommend prioritizing ongoing training and feedback to maintain proficiency in MI [31].

Participants had an opportunity to use every BCT within the mock client session, except for self-monitoring. The conversation veered away from self-monitoring toward goal setting, action planning, problem-solving, and social support due to the nature of the mock client’s script. This shift is significant because it highlights the adaptability and responsiveness of the kinesiologists in adjusting their approach based on the client’s needs and concerns (i.e., guiding client using MI). Although training may have improved kinesiologists taking the opportunity to use BCTs, more time may be required to enhance the quality of their use. In practice, kinesiologists have longer evaluation sessions, follow-ups, and one-on-one support, providing more time for implementing BCTs.

### Limitations and future research

One limitation of this study is the small sample size and non-probability convenience sampling, attributed to limited kinesiologists working at specific community-based organizations. However, significant t-tests with medium to large effect sizes highlight the promise of these modules. Although a control group and larger recruitment would have been optimal, their absence was due to the co-creation nature of the project. Regarding the COM-B questionnaire, only two items were used for each sub-component of the COM-B model to make it more feasible to assess each component of the modules (MI, BCTs, and APA). Although a more comprehensive assessment of each COM-B component would be ideal, the decision to use only two items was influenced by the applied setting in which the modules were delivered, where a longer questionnaire would have increased participant burden [32]. Although not feasible for this study, follow-up measurements of kinesiologists’ COM-B after a few months could have provided valuable insights into changes in their practice resulting from the training. Finally, another limitation is the low number of participants completing the mock client sessions, with longer sessions beyond 15 minutes needed for richer data. Future studies could explore the integration of these modules within organizations to understand adoption, use, and long-term effects on kinesiologists’ behaviours and members’ physical activity levels and use of BCTs.

## Conclusion

This study successfully co-created and evaluated training resources for kinesiologists at community-based APA programmes, enhancing their capability, opportunity, and motivation in utilizing BCTs, and APA prescription. Our process bridged an important theory-to-practice gap highlighted in the literature. The incorporation of two theories within a co-creation process highlights the conceptual and knowledge-based strengths of this study while addressing the existing gap and need for evidence-based tools. This study demonstrated the effectiveness of a feasible and usable training resources that will serve as valuable assets for community-based APA programme, thus fulfilling a dire need for this setting.

## Supplementary Material

ibae065_suppl_Supplementary_Material

## Data Availability

De-identified data from this study are not available in a public archive. De-identified data from this study will be made available (as allowable according to institutional IRB standards) by emailing the corresponding author.

## Welfare of Animals

This article does not contain any studies with animals performed by any of the authors.
