# Supplementary material for: Enhancing adapted physical activity training for community organizations: co-construction and evaluation of training modules
Source: Transl Behav Med. 2024 Nov 29;15(1):ibae065. doi: 10.1093/tbm/ibae065 (PMC11756304; doi:10.1093/tbm/ibae065)
Supplement: ibae065_suppl_Supplementary_Material [file ibae065_suppl_supplementary_material.docx]

**Supplemental Materials**

**Table A**

*Employed Strategies Ensuring Adherence to IKT Guiding Principles*

| Strategies Used | IKT Guiding Principles (Gainforth et al., 2021) |
| --- | --- |
| Creation of working group | 1: Partners develop and maintain relationships based on trust, respect, dignity, and transparency.  7: Partners address ethical considerations  8: Partners respect the practical considerations and financial constraints of all partners. |
| Working group meeting 1 and 2 discussing the needs of the Adaptavie and Viomax and the steps for creation of modules (including NGT, shareable whiteboard) | 2: Partners share in decision-making.  3: Partners foster open, honest, and responsive communication.  4: Partners recognize, value, and share their diverse expertise and knowledge.  5: Partners are flexible and receptive in tailoring the research approach to match the aims and context of the project. |
| Working group meeting 3, 4 and 5: editing the modules and discussing the contents of modules (including NGT, shareable whiteboard), results interpretation and knowledge mobilization plans | 2: Partners share in decision-making  3: Partners foster open, honest, and responsive communication.  4: Partners recognize, value, and share their diverse expertise and knowledge.  5: Partners are flexible and receptive in tailoring the research approach to match the aims and context of the project. |
| Sharing the IKT principles during each working group meeting | 1: Partners develop and maintain relationships based on trust, respect, dignity, and transparency.  3: Partners foster open, honest, and responsive communication. |
| Email communications for updates, steps to be taken, and shared decision-making. | 3: Partners foster open, honest, and responsive communication. |
| Evaluating the modules for kinesiologists employed at Adaptavie and Viomax | 6: Partners can meaningfully benefit by participating in the partnership.  7: Partners address ethical considerations.  8: Partners respect the practical considerations and financial constraints of all partners. |

*Note.* The integrated knowledge translation guiding principles for conducting research in partnership informed from Gainforth et al., (2021).

**Text A: Phase 1 Procedures and Analysis**

**Module Planning, Development and Refinement**

***Working Group Meetings 1 and 2.*** To identify the needs and adhere to Integrated Knowledge Translation (IKT) principles, the Nominal Group Technique (NGT) procedures were integrated into the first working group meeting allows group members to generate a high number of ideas, discuss ideas equally among members, and rank ideas attentively, thus increasing the quality of the discussion outcomes [22]. NGT allows for a clear identification of agreements and disagreements among members, facilitating detailed summaries and further feedback and discussion (Gallagher et al., 1993). NGT was used to guide the second working group meeting specifically in generating ideas and discussions, although ranking ideas and voting for a priority list were not included given the meeting's purpose (i.e., categorizing existing ideas into general themes).

***Module Creation.*** The nine training modules were created using Rise 360—an interactive module creation platform by Articulate. Five researchers from two universities were involved in the creation of the modules. The researchers have varying experiences in topics related to exercise psychology, kinesiology, APA prescription, and motivational interviewing. The modules include text, photos, audio, knowledge questions (multiple choice and written reflections), learning activities, and case studies. Features such as modality, and mode of delivery were in line with the training research that have used Kolb’s theory as an educational framework (Davitadze et al., 2022; Overwijk et al., 2022). The general topics of the modules were motivational interviewing, BCTs and adapted physical activity prescription. Motivational interviewing techniques included in the m open-ended questions, reflective listening, and choice provision (Miller & Rollnick, 2013). The training in Behavior Change Techniques (BCTs) involved teaching kinesiologists the meaning of the skills and how to apply them with persons with disabilities in a physical activity context.

***Working Group Meeting 3 and 4.*** To obtain usability feedback from working group meeting 3, each member individually reviewed the first module and noted any comments on the shared whiteboard link. When participants completed the module, they also answered the following questions to evaluate usability: “To what extent is this module feasible and useful for your kinesiologists? What do you think of the structure and format of the module? (Interactivity, clarity, aesthetics, level of difficulty)”. The second module was then reviewed as a group, where participants shared their comments as the module was presented on the shareable whiteboard. Prior to the fourth meeting, working group participants were sent a new module on a weekly basis as they were completed. Along with the modules, they received a shareable whiteboard link where they provided usability comments and feedback using the same questions discussed during the third working group meeting. The creation of each new subsequent module was informed and enhanced based on the usability comments from the first two modules, leading to the need for only one round of revisions from the working group. The comments were reviewed during the fourth meeting and any necessary changes were made to the modules.

**Analysis**

The results from the 10-item list generated by each member in the first working group meeting were summarized by the group organizer. The group was asked to identify the top 10 ideas and rank them in order of priority. Each idea was scored according to their rank, with 10 points given to the 1st item and 1 point for the 10th item. The importance that a person gives to each item was revealed and compared between group members.

The group collectively categorized the 10 ideas into two distinct themes. During the second working group meeting, the two themes were further sub-categorized into ideas for potential training modules. All members reached a collective agreement on the specific content to be included in the modules for each theme. The ideas addressed in the first working group meeting resulted with votes from each participant, leading to a collective vote of the final 10 ideas. The group collectively categorized the 10 ideas into two distinct themes. During the second working group meeting, the two themes were further sub-categorized into ideas for potential training modules. All members reached a collective agreement on the specific content to be included in the modules for each theme. The comments received on the sub-content ideas for each theme were extracted, summarized, and visually presented through tables and figures. The comments retrieved from the third and fourth working group meetings regarding module usability were grouped into four distinct categories. These categories were then used to make final edits and changes to each module.

**Figure A**

*Nominal Group Technique Procedures*

*Note.* Adapted from Gallagher, Hares [22].

**Table B**

*Feasibility of Modules*

| Module | Overall Mean (SD) | Appealing Mean (SD) | Relevance Mean (SD) | Language Mean (SD) | Specificity/ unambiguity Mean (SD) | Acceptability Mean (SD) | Adverse effects Mean (SD |
| --- | --- | --- | --- | --- | --- | --- | --- |
| 1.Introduction to MI | 6.56 (0.17) | 6.43 (0.51) | 6.36 (1.08) | 6.57 (0.65) | 6.79 (0.65) | 6.64 (0.50) | 1.46 (0.52) |
| 2.The Spirit of MI | 6.43 (0.24) | 6.07 (0.73) | 6.43 (0.76) | 6.64 (0.50) | 6.64 (0.50) | 6.36 (0.75) | 1.62 (0.87) |
| 3.The Process of MI | 6.37 (0.17) | 6.29 (0.61) | 6.36 (0.63) | 6.14 (0.86) | 6.50 (0.52) | 6.57 (0.51) | 1.62 (0.87) |
| 4.Self-monitoring | 6.23(0.26) | 5.79 (1.25) | 6.29 (0.73) | 6.36 (0.84) | 6.29 (0.73) | 6.43 (0.76) | 1.92 (1.50) |
| 5.Goal setting | 6.54 (0.22) | 6.21 (0.98) | 6.64 (0.63) | 6.79 (0.43) | 6.64 (0.50) | 6.43 (0.94) | 1.54 (0.88) |
| 6.Action Planning / Problem Solving | 6.41(0.22) | 6.21 (0.70) | 6.36 (0.63) | 6.71 (0.47) | 6.21 (1.12) | 6.57 (0.65) | 1.54 (0.88) |
| 7.Social Support | 6.50 (0.16) | 6.29 (0.47) | 6.57 (0.65) | 6.71 (0.47) | 6.50 (0.65) | 6.43 (0.65) | 1.46 (0.88) |
| 8.Adapted Physical Activity Prescription | 6.66 (0.19) | 6.64 (0.50) | 6.87 (0.36) | 6.71 (0.47) | 6.36 (0.75) | 6.71 (0.61) | 1.62 (0.96) |
| 9.Case Study | 6.56 (0.20) | 6.21 (0.70) | 6.71 (0.47) | 6.57 (0.51) | 6.64 (0.50) | 6.64 (0.63) | 1.46 (0.88) |

**Table C**

*Motivational Interviewing Utterances: Mock Client Sessions*

| Utterances | Mean | SD | Median | Minimum | Maximum | *n (used) |
| --- | --- | --- | --- | --- | --- | --- |
| Total Utterances | 34.33 | 3.91 | 34.0 | 31 | 44 | 9 |
| Giving Information | 5.00 | 2.60 | 4.00 | 2 | 10 | 9 |
| Total Questions | 14.78 | 5.33 | 17.0 | 4 | 21 | 9 |
| Open-ended Questions | 5.56 | 2.88 | 5.00 | 0 | 9 | 8 |
| Simple Reflections | 2.56 | 1.42 | 2.00 | 1 | 5 | 9 |
| Complex Reflections | 3.78 | 2.54 | 4.00 | 0 | 9 | 8 |
| Seeking Collaboration | 2.00 | 1.41 | 2.00 | 0 | 4 | 8 |
| Emphasizing Autonomy | 2.00 | 1.41 | 2.00 | 0 | 5 | 8 |
| Affirming | 1.11 | 1.17 | 1.00 | 0 | 4 | 8 |
| Persuade with Permission | 3.11 | 1.69 | 4.00 | 0 | 5 | 8 |
| Persuade | 1.67 | 1.73 | 1.00 | 0 | 5 | 6 |

*Note.* n = 9. *n (used) indicates the number of participants who used at least one utterance.

**Figure B**

*Mock Client Behaviour Change Technique Coding Sheet*


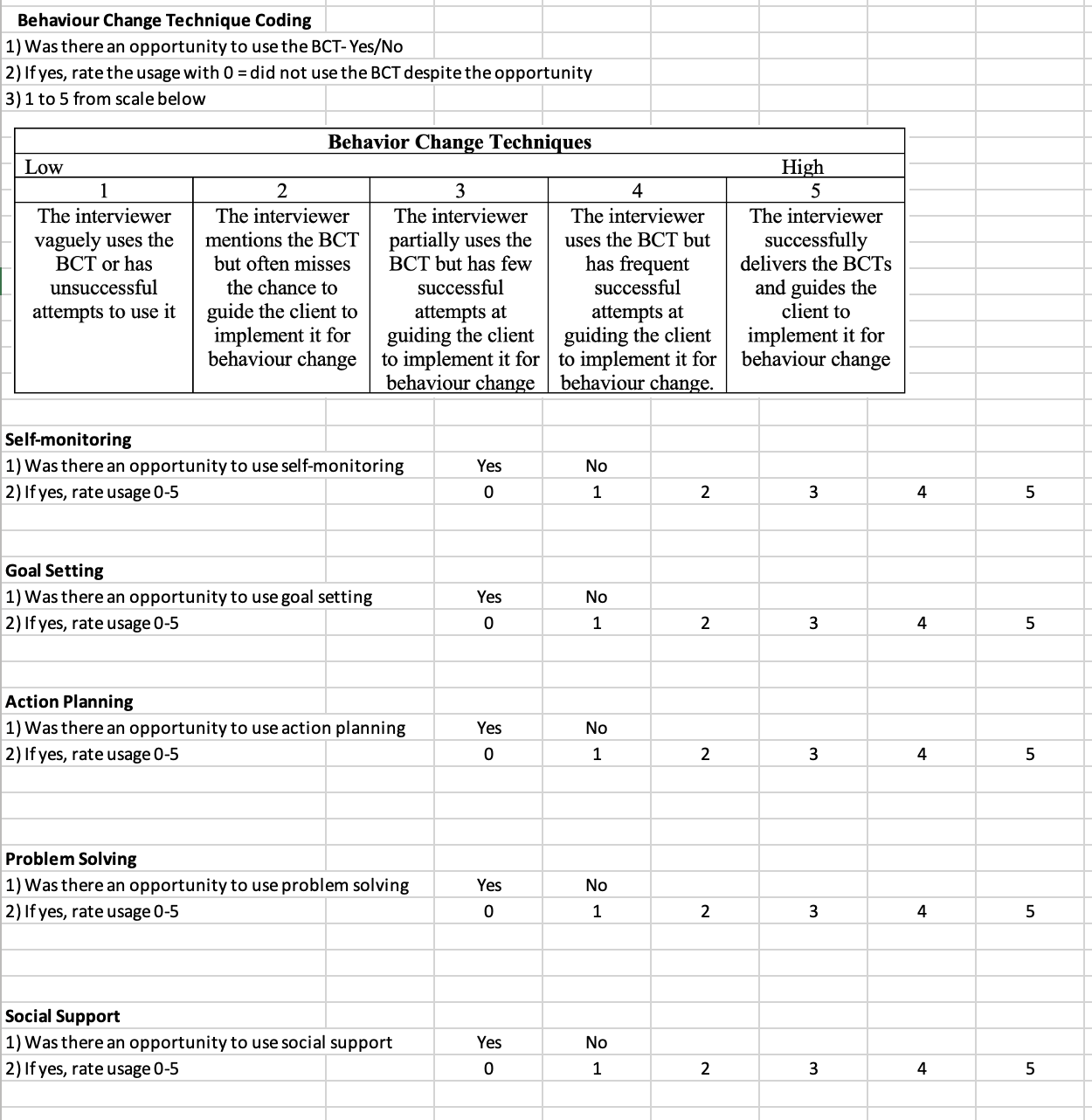


*Note.* Behaviour change techniques coding sheet and quality scale for mock client sessions.
